# MR Thermometry Accuracy and Prospective Imaging-Based Patient Selection in MR-Guided Hyperthermia Treatment for Locally Advanced Cervical Cancer

**DOI:** 10.3390/cancers13143503

**Published:** 2021-07-13

**Authors:** Iva VilasBoas-Ribeiro, Sergio Curto, Gerard C. van Rhoon, Martine Franckena, Margarethus M. Paulides

**Affiliations:** 1 Department of Radiotherapy, Erasmus MC Cancer Institute, University Medical Center Rotterdam, 3015 GD Rotterdam, The Netherlands; s.curto@erasmusmc.nl (S.C.); g.c.vanrhoon@erasmusmc.nl (G.C.v.R.); m.franckena@erasmusmc.nl (M.F.); m.m.paulides@tue.nl (M.M.P.); 2 Department of Radiation Science and Technology, Faculty of Applied Sciences, Delft University of Technology, 2629 JB Delft, The Netherlands; 3 Center for Care and Cure Technologies Eindhoven (C3Te), Department of Electrical Engineering, Eindhoven University of Technology, 5600 MB Eindhoven, The Netherlands

**Keywords:** MR thermometry, PRFS, RF hyperthermia, locally advanced cervical cancer, accuracy, bias, precision, imaging-based selection

## Abstract

**Simple Summary:**

Monitoring and controlling the temperature distribution combined with precise energy delivery are key components for hyperthermia treatment success. Magnetic resonance (MR) imaging is used clinically to monitor the temperature of the treated volume non-invasively. However, there are no comprehensive systematic studies on MR thermometry accuracy during deep pelvic hyperthermia, and the few investigational studies suffer from a high probability of bias due to lacking objective criteria for data inclusion. This study presents the first systematic analysis and defines an imaging-based criterion for prospective patient selection to standardize clinical MR thermometry accuracy assessments.

**Abstract:**

The efficacy of a hyperthermia treatment depends on the delivery of well-controlled heating; hence, accurate temperature monitoring is essential for ensuring effective treatment. For deep pelvic hyperthermia, there are no comprehensive and systematic reports on MR thermometry. Moreover, data inclusion generally lacks objective selection criteria leading to a high probability of bias when comparing results. Herein, we studied whether imaging-based data inclusion predicts accuracy and could serve as a tool for prospective patient selection. The accuracy of the MR thermometry in patients with locally advanced cervical cancer was benchmarked against intraluminal temperature. We found that gastrointestinal air motion at the start of the treatment, quantified by the Jaccard similarity coefficient, was a good predictor for MR thermometry accuracy. The results for the group that was selected for low gastrointestinal air motion improved compared to the results for all patients by 50% (accuracy), 26% (precision), and 80% (bias). We found an average MR thermometry accuracy of 2.0 °C when all patients were considered and 1.0 °C for the selected group. These results serve as the basis for comprehensive benchmarking of novel technologies. The Jaccard similarity coefficient also has good potential to prospectively determine in which patients the MR thermometry will be valuable.

## 1. Introduction

Several randomized clinical studies have shown the benefit of hyperthermia as a sensitizing agent for chemotherapy and/or radiotherapy [1,2,3,4,5,6,7,8]. Franckena et al. showed a statistically significant correlation between thermal dose delivered during treatment and treatment outcome in a group of patients with locally advanced cervical cancer (LACC) [9]. This study suggests that the quality of hyperthermia treatment delivery is crucial to its clinical success. Hence, the 3D temperature distribution knowledge, supplemented with equipment that facilitates precise and adaptive delivery, is critical for improving treatment outcome [10,11,12]. Magnetic resonance (MR)-guided hyperthermia is considered the most promising technological platform to monitor the tumor and healthy tissue temperature non-invasively for real-time dose-optimization and dosimetry in multi-institution clinical trials [13,14,15,16,17]. However, systematic assessment of the accuracy and reliability of the non-invasive 3D-MR temperature measurements during treatment is currently lacking. At the same time, this is crucial for deciding on clinical acceptance and for benchmarking technology improvements.

Temperature measurements during hyperthermia treatment can be performed by direct thermometry using invasive and/or intraluminal thermometry probes or by indirect methods such as MR thermometry. Intraluminal thermometry is currently the gold standard for temperature assessment during the treatment of patients with LACC [18,19]. This method will remain the gold standard until MR thermometry presents enough accuracy to provide absolute temperature information. In this technique, temperature probes are inserted into closed-tip catheters previously positioned in body cavities. Invasive/intraluminal thermometry presents severe limitations [20] since it samples only data at a specific location and/or along the implanted catheter, leading to a limited spatial resolution. Additionally, this technique is unpleasant for the patient and can be associated with possible risks such as hemorrhages or infections [19,21,22,23]. MR thermometry offers the advantage that it can non-invasively monitor temperature changes in the treated volume and surrounding tissues. Additionally, it offers the possibility to characterize treatment efficacy by observing the required thermal metrics in real time during treatment. This technique brings opportunities for dynamic treatment delivery feedback control [24,25,26,27,28,29,30], as well as treatment planning validation [13,17,31], and assessment of thermoregulation in tissues [32,33,34,35]. There are several MR thermometry methods; the proton resonance frequency shift (PRFS) method is the most widely used due to its linearity and sensitivity [36,37,38,39,40]. 

Interest in MR-guided hyperthermia systems has steadily grown over the last decades [14,16,41,42]. Several studies have demonstrated the feasibility of non-invasive MR thermometry and benchmarked MR thermometry against invasive/intraluminal thermometry. Gellermann et al. [43] showed the potential of MR thermometry in patients with recurrent rectal carcinoma. They found a correlation of R^2^ = 0.67 and an accuracy of 1.5 °C between MR thermometry and thermistor probe readings. A follow-up study included patients with soft tissue sarcomas of the lower extremities and pelvis [44] and showed a correlation of R^2^ = 0.96 between MR thermometry and thermistor probe readings. Craciunescu et al. [45] evaluated the bias between MR thermometry and invasive thermometry for high-grade extremity soft-tissue sarcomas. They found that the mean differences in a small volume of interest around interstitial probe positions were below 1 °C. However, Craciunescu et al. [45] showed that in regions at muscle/fat or tumor/fat, the bias was 1.89 °C. For large extremity soft tissue sarcoma, Stauffer et al. [46] showed that the bias between MR thermometry and interstitial measurements was 0.85 °C. In a more recent study, Unsoeld et al. [33] found a correlation between MR thermometry data and pathologic response for soft-tissue sarcomas of the lower extremities. Assessment of MR thermometry performance in deep pelvic tumors; i.e., nearby inner patient locations with motion such as moving air in the intestines has been evaluated in only a few studies [43,44,47]. These studies did show a qualitative correlation between invasive/intraluminal probe measurements and MR thermometry. However, these did not evaluate the accuracy in a volume of interest close to the temperature probes, the latter being crucial information for clinical acceptance. In addition, no studies have reported MR thermometry temporal precision for RF hyperthermia [48]. Finally, replicating these results is lacking and will be cumbersome in a retrospective setting due to the strong but not clearly defined patient selection. Hence, the accuracy of MR thermometry during deep pelvic hyperthermia treatments remains ambiguous. 

In this study, we investigated MR thermometry in patients with LACC and assessed accuracy, temporal precision, and bias. As the gold standard, we used intraluminal placed temperature probes. In addition, we investigated the feasibility of a standardized pre-treatment patient selection based on a relevant and measurable imaging parameter. 

## 2. Materials and Methods

### 2.1. Patients and Clinical Protocol

This study included 14 patients diagnosed with locally advanced cervical carcinoma. All patients were treated at the Erasmus Medical Center with curative intent using hyperthermia as an adjunct to radiotherapy. Approval of the medical ethics committee was obtained prior to start of the study (MEC 2015-108). The patient/tumor characteristics are presented in Table 1. 

All patients were treated in the BSD-2000-3D MR-compatible system (Pyrexar Medical Corp., Salt Lake City, UT, USA) [14] integrated into a 1.5 T GE Signa Excite scanner (General Electric Healthcare, Waukesha, WI, USA). Following the non-MR monitored procedure, intraluminal thermometry was acquired during the hyperthermia treatment by Bowman probes inserted into closed tip catheters placed in the bladder, vagina, and rectum before the hyperthermia treatment. Temperature mapping along the catheters was performed every 5 min with a step size of 1 cm and a maximum mapping length of 14 cm. Each patient received, on average, three hyperthermia treatments of approximately 90 min within the BSD-2000-3D MR-compatible hyperthermia system during the entire course of radiotherapy (Table 1). A patient-specific treatment plan was delivered for each patient. Treatment settings for power and phase were adjusted accordingly following patient complaints and/or if healthy tissue temperature exceeded 43 °C [49,50].

### 2.2. MR Thermometry Image Acquisition

**Figure 1:**
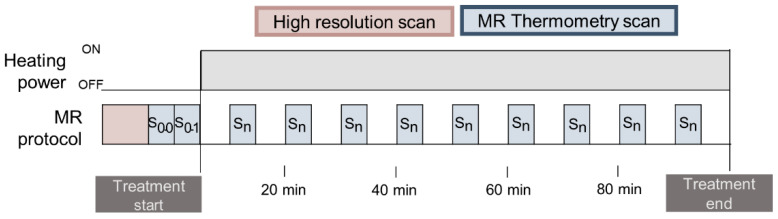
Description of the clinical MR protocol. Each sequence is represented with a color. High-resolution scan and two MR thermometry scans were taken before the treatment, and approximately nine MR thermometry scans were performed during treatment.

The schematic description of the MR protocol is presented in Figure 1. We used two types of scans: the high resolution scan for verifying the patient positioning and anatomic information, and the MR thermometry scan for temperature imaging [17]. 

For temperature monitoring, we employed the PRFS method [36,40,51]. The clinical sequence provided by the manufacturer is the double echo gradient recalled echo (DEGRE) sequence [36,43,44,47] with parameters: echo times: TE = 4.8 and 19.1 ms; repetition time: TR = 620 ms; 25 axial slices; slice thickness = 1 cm; field of view (FOV) = 50 × 50 cm; acquisition matrix = 128 × 128; reconstruction matrix = 256 × 256; flip angle = 40°; scan time = 83 s. Each MR thermometry scan (S) presents 25 phase images (φ) and 25 magnitude images.

### 2.3. Contouring and Region Selection

**Figure 2:**
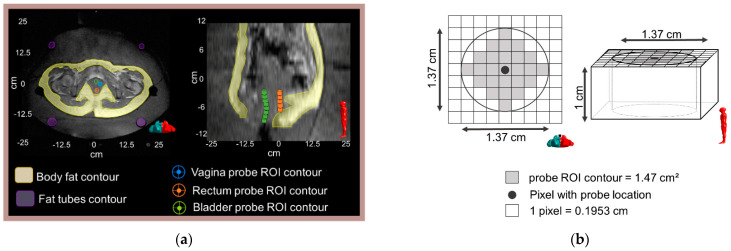
(**a**) An axial and a sagittal T1-weighted MR image of the pelvic are shown along with an illustration of the location of the Bowman probes points and the corresponding delineated ROIs: vagina, rectum, and bladder. The left and right images correspond to the middle axial slice (z = −2 cm) and middle sagittal slice (x = 0 cm), respectively. Body fat and fat-like tube ROIs are indicated in the axial MR image. (**b**) Schematic representation of the probe ROI contour in the axial and sagittal view.

**Figure 3:**
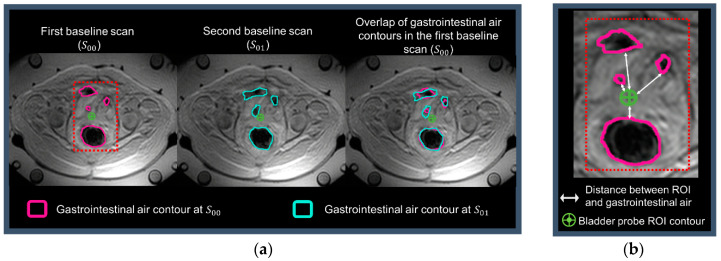
The axial anatomic images and a zoomed region from MR thermometry reference scans are shown: (**a**) presents the gastrointestinal air contours in the two first baseline scans and the overlap of these contours in the first baseline scan; (**b**) shows the distances between the probe and gastrointestinal air contour. This representative treatment session presented: Jaccard coefficient = 0.67; minimum distance = 0.2 cm; fat volume = 8782.7 mL; and gastrointestinal air volume = 548.5 mL.

T1-weighted datasets (high-resolution scan in Figure 1) were used to identify the catheters containing Bowman probes and delineate regions of interest. From the 25 slices of the T1-weighted datasets, only two to eight slices were used to identify intraluminal locations due to the limited probe range. Figure 2a presents an example of the probe range in the bladder and rectum, where, in the sagittal view, the rectum probe was identified in only in five slices (−2 cm to −7 cm), while the bladder probe was identified in eight slices (−2 to −10 cm). Note that the probe located in the vagina is not visualized in this sagittal image. Pointwise matching the probes to the MR temperature mapping was difficult; therefore, we draw a circular region of interest (ROI) for each location identified to reduce the impact of spatial mismatches. As shown in Figure 2b, the ROIs had a diameter of 1.37 cm, resulting in an ROI area of 1.47 cm^2^. Body fat was delineated for MRT correction purposes and to evaluate its impact on MR thermometry accuracy. As presented in Figure 3, gastrointestinal air was delineated in the baseline scans to evaluate the impact of air volume and motion on MR thermometry accuracy during the treatment.

### 2.4. MR Thermometry Processing 

The PRFS method measures relative temperature differences (∆T) based on phase changes (∆φ) of the different MR thermometry scans. Hence, before the application of RF power, reference phase data ($\varphi_{00}$ and $\varphi_{01}$) was acquired to establish baseline temperature conditions. After power on, on average, nine phase datasets were acquired during the treatment session ($\varphi_{n})$. 

The acquired images were processed as described in the following steps:

1. **Uncorrected MR thermometry maps**: MR thermometry maps were calculated by taking the difference between the phase maps ($\varphi_{00}$ and $\varphi_{n}$), which is formulated as:
  (1) $$∆T(n)=\frac{\varphi_{n}{-\;\varphi }_{00}}{{\gamma \alpha B}_{0}\mathrm{TE}}\;$$
  where γ is the gyromagnetic ratio and equal to 267.5 × 10^6^ rad/T∙s; α is the PRF change coefficient, which is equal to −0.001 ppm/°C; $B_{0}$ is the magnetic field strength equal to 1.5 T; TE is the echo time equal to 19.1 ms; and n is the scan time.
2. **Low SNR masking**: For each MR thermometry map, voxels with low SNR corresponding to a temperature deviation > 3 °C with respect to three-by-three neighbors were masked to prevent the inclusion of noisy data in the voxels used for drift correction.
3. **B0 drift correction**: In addition to the four fat-like tubes included in the hyperthermia device, body fat (Figure 2) was used to compensate for changes of the static magnetic field B0. A 2D polynomial spatial-temporal correction was applied across the MR temperature maps such that temperature changes are reversed to zero in the selected fat regions. Hence, $T_{\mathrm{MR}}$ denotes the final corrected MR thermometry.
4. **Inaccurate data exclusion**: Unrealistic data was removed to avoid pollution by data points affected by confounders such as moving air or other motion. The absolute difference between intraluminal measurements and average MR thermometry measurement within ROIs was minimized. The absolute difference between the two measurements is given by Equation (2), and the minimization is given by Equation (3). The threshold for removal was found to be 7 °C, which was iteratively found between 0 °C and 20 °C using an optimization cycle.
  (2) $$G\left(p\right)=\left|\;\left(\frac{1}{\mathrm{card}\left(J\right)}\overset{j}{\underset{j=1}{\sum }}{\;T}_{\mathrm{MR}}{(n\;,\;p)\;}_{j}\right)-{\bar{T}}_{{\mathrm{probe}}_{\mathrm{ROI}}}\left(n\right)\;\right|$$
  (3) $$\mathrm{threshold}=\arg\underset{p}{\min}\;G(p)\;\mathrm{subject}\;\mathrm{to}\;0\;\leq \;p\;\leq \;20$$
  where ${\;T}_{\mathrm{MR}}(n)\;$ is the MR thermometry temperature; n is the scanning time; ${\bar{T}}_{{\mathrm{probe}}_{\mathrm{ROI}}}$ is the average intraluminal temperature along the catheter at each location (bladder, rectum, and vagina), j is the index of filtered voxels, and card(J) is the number of voxels within the ROI that were taken into account after applying the threshold.
5. **Average MR thermometry measurement within ROI**: For each probe location, at each scanning time, the average temperature was calculated within the delineated ROIs $({\bar{T}}_{{\mathrm{MR}}_{\mathrm{ROI}}}$ ). The formulation of the average temperature is given by:
  (4) $${\bar{T}}_{{\mathrm{MR}}_{\mathrm{ROI}}}\left(n\right)=\frac{1}{\mathrm{card}\left(J\right)}\overset{j}{\underset{j=1}{\sum }}{\;T}_{\mathrm{MR}}\left(n\right){\;}_{j}\;$$
  where ${\;T}_{\mathrm{MR}}(n)\;$ is the MR thermometry temperature, n is the scanning time, j is the index of voxels, and card(J) is the number of voxels within the ROI. Hence, for each intraluminal location and at each scan, a ${\bar{T}}_{{\mathrm{MR}}_{\mathrm{ROI}}}$ was calculated (${\bar{T}}_{{\mathrm{MR}}_{\mathrm{ROI}:\mathrm{rectum}}}$, ${\bar{T}}_{{\mathrm{MR}}_{\mathrm{ROI}:\mathrm{bladder}}}$ , ${\bar{T}}_{{\mathrm{MR}}_{\mathrm{ROI}:\mathrm{vagina}}}$).

### 2.5. Imaging-Based MRT Accuracy Prediction Parameters

The PRFS method measures the relative temperature, which is acquired by the subtraction of temporal phase maps. The supposition behind this method is that the anatomy is stationary, and any phase change between the subtracted images results entirely from temperature changes. Therefore, motion leads to temperature errors, i.e., the intra-scan motion leads to motion-dependent measurement blurring, and inter-scan motion leads to misregistration with the reference phase image. To enable prospective patient selection, we formulated four imaging-based parameters to associate them with MR thermometry accuracy. These were fat volume, gastrointestinal air volume, gastrointestinal air motion, and the minimum distance between the gastrointestinal air contours and the probe ROIs. The minimum distance used consisted of the mean minimum distance calculated in all slices where probe ROIs were delineated (Figure 3b). 

As shown in Figure 3a, for each treatment session, the gastrointestinal air was delineated using the magnitude of the baseline scans ($S_{00}\;$ and $S_{01}$). $S_{00}\;$ was used to quantify the initial gastrointestinal air volume, and $S_{00}\;$ and $S_{01}$ were used to quantify gastrointestinal air motion. The average time between the start of the two baseline scans within all treatment sessions was 97 ± 10 s. Note that an MR thermometry scan duration is 83 s, so the average time between the end and start of the two scans was 14 s. Gastrointestinal air motion was measured using the Jaccard similarity coefficient [52,53,54]:

(5) $$J\left(A,\;B\right)=\frac{\left|A\;\cap \;B\right|}{\left|A\;\cup \;B\right|}\;$$

where A and B are the air contours in $S_{00}\;$ and $S_{01}$, respectively (Figure 3). This coefficient is somewhat equivalent to the Dice similarity coefficient but is more sensitive to the absence or presence of overlap. Even though these coefficients are monotonic to one another, the Jaccard coefficient tends to penalize contour differences more than the Dice similarity. Moreover, a higher Jaccard coefficient indicates better agreement between contours. Additionally, the minimum distance between gastrointestinal air and probe ROI contour was computed, as illustrated by the arrows in Figure 3b.

The receiver operating characteristic (ROC) curve was used to analyze how predictive each feature is for acceptable/reliable MR thermometry, and to determine the cut-off values [55,56]. MR thermometry was classified as acceptable for each treatment session (true-condition) when in-accuracy was equal or lower than 1 °C [57], and false otherwise. The area under the ROC curve (AUC) was used as a robust measure to evaluate the performance of the score classifier [58,59]. The AUC ranges from 0.5 (random predictive ability) to 1.0 (perfect predictive ability). The optimal cut-off value ($C_{\mathrm{opt}}$) was obtained by taking the minimum distance from the ROC curve to the top-left corner or point (0,1) [60]. In addition, the 95% confidence interval was calculated for each feature and the *p*-value for the null hypothesis that the AUC is equal to 0.5 (random relation).For each treatment session, the accuracy of MR thermometry measurements (Equation (6)) provided the degree of closeness of the measured temperature change to the actual temperature change (i.e., intraluminal temperature) [48,61]. Given the importance of keeping the proper heating range, we consider the accuracy of ≤1 °C as suitable [48,57]. The temporal precision was determined by the variability of the spatial mean temperature in an ROI across all time points, shown in Equation (7). Since precision provides the reproducibility and repeatability of measurements, we consider that precision should be ≤1 °C [57]. We calculated the bias as the mean error between MR thermometry and intraluminal measurements (Equation (8)) [48,61]. This parameter shows if there is an over or underestimation of temperature. We consider a bias of ≤|0.5 °C| as appropriate [48]. For each intraluminal location (bladder, rectum, and vagina), we described the precision, accuracy, and bias measurements by mean (μ) ± standard deviation (σ). The accuracy, precision, and bias at each probe location were compared using one-way ANOVA analysis [62].

(6) $$\mathrm{Accuracy}=\frac{1}{n}\;\sum_{j=1}^{n}\;\left|{\bar{T}}_{{\mathrm{MR}}_{\mathrm{ROI}},\;j}\;-{\bar{T}}_{{\mathrm{probe}}_{\mathrm{ROI}},\;j}\right|\;$$

(7) $$\mathrm{Precision}=\mathrm{std}\;(\;\frac{1}{n}\;\sum_{j=1}^{n}{\bar{T}}_{{\mathrm{MR}}_{\mathrm{ROI}},\;j}\;)\;$$

(8) $$\mathrm{Bias}=\frac{1}{n}\;\sum_{j=1}^{n}{\bar{T}}_{{\mathrm{MR}}_{\mathrm{ROI}},\;j}\;-{\bar{T}}_{{\mathrm{probe}}_{\mathrm{ROI}},\;j}\;,$$

where ${\bar{T}}_{{\mathrm{MR}}_{\mathrm{ROI}},\;j}$ is the average MR thermometry in the ROI, ${\bar{T}}_{{\mathrm{probe}}_{\mathrm{ROI}},\;j}$ is the average intraluminal temperature measured along the catheter, and n are measured time points.

Furthermore, for each evaluation parameter, the average deviation from the acceptable threshold was calculated. In other words, the accuracy, precision, and bias within the three locations was calculated and compared with the acceptable threshold to acquire the deviation.

## 3. Results

### 3.1. Predictive Value for MRT Accuracy of Imaging-Based Parameters

**Figure 4:**
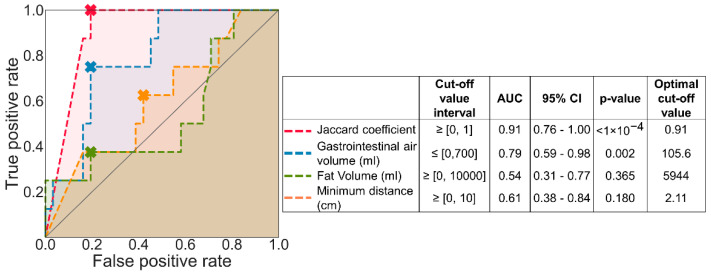
ROC curve analysis of each predicted condition. The cross marked in each curve indicates the optimal cut-off value, and the shade under each represents AUC. The identity line presented by a solid grey line represents the ROC curve with an AUC equal to 0.5.

Figure 4 reports the ROC analysis for the different patient features concerning the predictive value for acceptable MR thermometry accuracy. Gastrointestinal air motion (Jaccard coefficient) outperformed the AUC value and significance and had an optimal cut-off value of 0.91 for a scanning interval of 97 s. The AUC score of gastrointestinal air volume was considerably high (0.79), and the optimal cut-off values were equal to 105.6 mL. The fat volume and minimum distance presented an AUC indicating that these parameters should be considered as random; i.e., they are not suitable predictors for acceptable MR thermometry.

**Figure 5:**
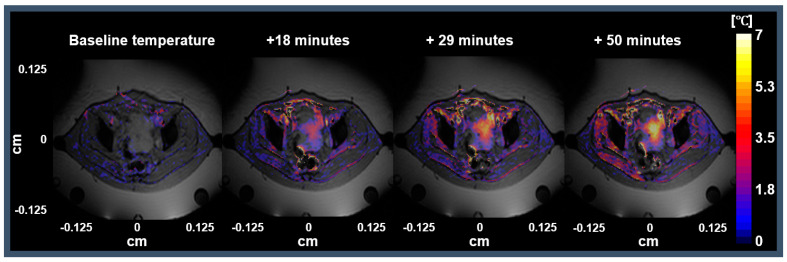
Representative session with Jaccard coefficient equal to 1 and the gastrointestinal air volume was equal to 221 mL. The first image represents the MR thermometry map between the phase images of $S_{00}$ and $S_{01}$. The following three images represent the MR thermometry maps after 18, 29, and 50 min from when RF power was applied (treatment start).

Figure 5 presents an example of a session where a Jaccard coefficient was equal to 1, and the gastrointestinal air volume was equal to 221 mL. The session exemplified in Figure 5 represents one of the 15 sessions where we observed that the air motion presented a higher impact than air volume. Hence, the Jaccard coefficient was considered the most suitable parameter for predicting the MR thermometry accuracy and, consequently, it was the parameter used for the treatment session selection.

### 3.2. MRT Accuracy for All Data versus MRT Accuracy from Selected Sessions

**Figure 6:**
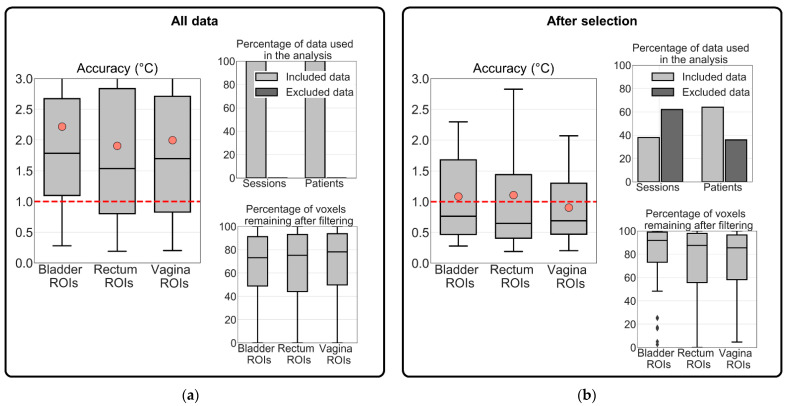
Evaluation of MR thermometry accuracy in (**a**) all treatment sessions and (**b**) in the group of selected treatment sessions. The MR thermometry was calculated for the three intraluminal locations. The evaluation of MR thermometry was based on the voxels remaining after filtering and data used in the analysis. The dashed red line represents the acceptable mean accuracy threshold (1 °C) and, in light red circles, the mean MR thermometry accuracy in each location. The inter-quartile range denotes the middle 50% of the dataset. The top box shows 75% of the dataset that falls below the upper quartile, while the bottom line consists of 25% of the dataset that falls below the lower quartile. The middle line represents the median value, and the line extending from the box represents 2.5% and 97.5% limits of the dataset.

**All data**: The robustness of MR thermometry accuracy prediction was evaluated by quantifying the temperature accuracy for all probe locations (Figure 6). Figure 6a shows that the median accuracy within the ROIs for all probe locations was 1.7 °C. Light red circles in Figure 6a mark the mean accuracy expressed in Table 2. The mean MR thermometry accuracy in all intraluminal locations was outside the acceptable threshold of 1 °C [48,57]. In addition, the total mean accuracy was equal to 2 °C. The differences in accuracy and the number of voxels used between the different intraluminal locations were insignificant (*p*-value > 0.05). 

**After selection**: The MR thermometry accuracy of the selected sessions (Jaccard coefficient ≥ 0.91) was lower than when considering all data. The median MR thermometry accuracy for the bladder, rectum, and vagina was 0.8 °C, 0.6 °C, and 0.7 °C, respectively (Figure 6b). The marked points in Figure 6b show that even though there was an improvement, the mean MR thermometry accuracy was within the acceptable values only in the vagina ROIs (0.9 °C). In contrast, the mean accuracy was equal to 1.1 °C in the bladder and rectum ROIs. Imaging-based selection excluded 36% of the total patients, but the percentage of voxels remaining after filtering increased from 76% to 88%. The differences in accuracy between the different intraluminal locations were not significant (*p*-value > 0.05). Note that significantly more voxels of the bladder ROIs remain after filtering than in the ROIs of the rectum and vagina (*p*-value = 0.04). 

**Figure 7:**
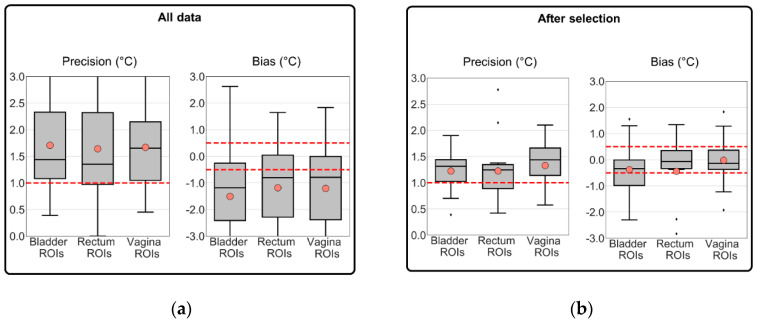
Evaluation of MR thermometry precision and bias in (**a**) all treatment sessions and (**b**) in the group of selected treatment sessions. The MR thermometry was calculated for the three intraluminal locations. The evaluation of MR thermometry was based on the voxels remaining after filtering and data used in the analysis. The dashed red line represents the acceptable mean accuracy threshold (1 °C) and, in light red circles, the mean MR thermometry accuracy in each location. The inter-quartile range denotes the middle 50% of the dataset. The top box shows 75% of the dataset that falls below the upper quartile, while the bottom line consists of 25% of the dataset that falls below the lower quartile. The middle line represents the median value, and the line extending from the box represents 2.5% and 97.5% limits of the dataset.

Table 2 summarizes the mean (µ) and standard deviation (σ) of MR thermometry accuracy, precision, and bias. These parameters were calculated for two datasets: all the treatment sessions and treatment sessions selected were based on gastrointestinal air motion (Jaccard coefficient). Table 2 presents the number and percentage of treatment sessions and patients resulting from each exclusion combination. MR thermometry accuracy, precision, and bias were acceptable when the air motion was used as the selection criterion based on the recommended thresholds. As presented in Table 2, for the selected dataset, the average accuracy and bias within all locations were equal or better than the acceptable threshold (Figure 7b), while the average precision was above the threshold (Figure 7b). In other words, the deviations for accuracy and bias were 0.0 °C and −0.3 °C, respectively, while for precision, the deviation was +0.2 °C. In comparison with including all sessions, we observed that the selection based on the Jaccard coefficient improved MR thermometry accuracy by 50%, precision by 26%, and bias by 80%. 

Figure 7 presents the additional evaluation of MR thermometry regarding precision and bias in all data and the selected dataset. In the selected dataset, the mean values for bias in the bladder, rectum, and vagina location were −0.4 °C, −0.4 °C, and 0.0 °C, i.e., all within the defined threshold value of ±0.5 °C [48]. Figure 7b shows that most of the selected sessions presented an MR thermometry bias within the limits. We observed that the most significant improvement was in the vagina ROIs (98%). In all and selected data, we observed an overall bias of −1.3 °C and −0.3 °C, respectively. These results indicate a general underestimation by MR thermometry. The average precision was approximately 1.3 °C for the intraluminal locations in the selected data, which is slightly above the required threshold (1 °C [57]). Note that the differences between the three intraluminal locations for precision and bias were not significant, both for all data and the selected data (*p*-value > 0.05). 

## 4. Discussion

### 4.1. Image Parameters to Select Treatments with Robust MRT

This study evaluated MR thermometry accuracy, precision, and bias before and after imaging-based patient selection. This evaluation compared MR thermometry in small ROIs with intraluminal measurements in the bladder, rectum, and vagina. Note that these were initially created to include the monitoring region of the Bowman probes. These regions were carefully identified for each location in the MR thermometry measurements (time and location) and compared with the gold standard, i.e., intraluminal temperature measurements. We observed that MR thermometry accuracy was poor and above the values (≥1 °C [48,57]) defined as acceptable when using all treatment sessions in the evaluation. We tested four patient-dependent features—fat volume, gastrointestinal air volume, gastrointestinal air motion, and the minimum distance between the gastrointestinal air contours and the probe ROIs. We used the ROC analysis to evaluate which features were predictors for MR thermometry reliability (Figure 4). Our analysis showed that gastrointestinal air motion was predictive for MR thermometry accuracy (AUC = 0.91). 

The accuracy of the PRFS method to acquire non-invasive temperature is strongly variable in the pelvic region because of the adjacent intestines and rectum. These organs often contain moving air and, consequently, causing significant susceptibility artifacts [33,44,51,63], which the PRFS method misinterprets as temperature changes. When using all the patient data, we found an average accuracy of 2.0 °C (Figure 6a, Table 2). By selecting treatment sessions based on the amount of air motion between the two baseline images, we could identify which sessions would present improved MR thermometry accuracy compared to all treatment sessions. In addition, we observed an acceptable MR thermometry precision and bias since we found a mean value equal to 1.2 °C and −0.3 °C, respectively (Table 2 and Figure 7). In comparison to all patient data, MR thermometry precision and bias improved 26% and 80%, respectively. Overall, we presented and validated a selection criterion based on an imaging parameter that can be used prospectively to ensure reliable MR-thermometry measurements. 

Previous studies have evaluated the MR thermometry accuracy and bias; however, no clinical studies have reported MR thermometry temporal precision. The study conducted by Dadakova et al. [47] reported MR thermometry accuracy and bias equal to 0.40 °C and 0.04 °C, respectively. This study included a group of patients with myxoid liposarcoma (one patient), mucinous rectal cancer (one patient), and rectal adenocarcinoma (two patients). The results found in this study were within the acceptable thresholds; however, the measurement regions were further away from internal motion. One of the patients was excluded from the analysis because of artifacts caused by the air in the rectum. For rectal carcinoma, the study carried out by Gellermann et al. [43] showed that MR thermometry accuracy was equal to ±1 °C after 20 min of treatment time and increased during the treatment to ±1.5 °C. Gellermann et al. [44] found an MR thermometry bias equal to 1.1 °C between several ROIs delineated in the water of water bolus and the probe measurements. Moreover, in both studies, MR thermometry accuracy and bias were not measured in the small volumes where the probes are located and, hence, are prone to underestimate the inaccuracies. 

In addition to the B0 drift correction, we applied a filtering process to the MR thermometry maps to remove unrealistic data. The threshold found and used was equal to 7 °C, which implies that the maximum absolute temperature found was approximately 44 °C. Several studies have shown that the increase in the tumor site temperature can be higher than 7 °C [33,34,43,44,45,47]. This threshold was optimized based on the agreement between MR thermometry and intraluminal measurements. Therefore, the tumor site was not taken into account since intraluminal measurements indicate tumor temperature but hardly ever within the tumor. Additionally, we found that many more data points remained valid after the filtering process in the selected group of patients than all patients/treatment sessions (Figure 6). This result indicates that our filtering process also removes systematic errors and that the excluded treatment sessions indeed incorporated corrupt or noisy data.

Table 3 presents the average temperature increase measured at each location and the temperature increase from two studies by Gellermann et al. [43,44]. For rectal carcinoma, the high average measured temperatures in the bladder (>7 °C) were reported and were explained as due to the anatomic changes caused by the bladder filling during treatment [43]. At Erasmus MC, a transurethral catheter was positioned inside the patient’s bladder, so anatomy changes due to bladder filling were expected to be less likely to occur during treatment. In contrast to Gellermann et al. [43], our results show that the temperature increase in the bladder was approximately the same between MR thermometry and intraluminal measurements (Table 3). As shown in Table 3, for soft tissue sarcomas, Gellermann et al. [44] found a higher average increase of temperature from MR thermometry measurements than intraluminal measurements. However, that study suggests that MR thermometry measurements overestimate the temperature by 1 °C to 2 °C. In the studies mentioned, treatment sessions were removed from the analysis due to disturbances caused by technical reasons, incomplete MR datasets, and/or restlessness of the patient. Our study used fewer patient data (64%) compared to the two studies (100%). Regarding the percentage of treatment sessions taken into account, our study used more treatment sessions (38%) compared to Gellermann et al. [43] (20%), but less compared to Gellermann et al. [44] (50%). Overall, our results showed a good agreement between intraluminal and MR thermometry measurements (0.1 °C to 0.4 °C) in the selected data. 

### 4.2. Clinical Relevance

In general, this study shows the feasibility of MR thermometry in the pelvic region and indicates directions to improve MR-thermometry for hyperthermia treatment of patients with LACC. We retrospectively looked at several imaging parameters and evaluated MR thermometry accuracy based on these. We considered using imaging parameters (gastrointestinal air motion: Jaccard coefficient) useful, not only as a selection criterion but also as tools to further improve hyperthermia treatment planning. Hyperthermia treatment planning is designed to predict and optimize hyperthermia treatment performance. One of the steps from this procedure is to segment different tissues to generate a 3D patient model. In this study, we selected patients prospectively using air motion between two anatomical images taken before the treatment. Since gastrointestinal air is one of the tissues being segmented, the Jaccard coefficient could be calculated during the treatment planning phase, which would provide valuable information about the expected reliability of MR thermometry. 

At Erasmus MC, deep regional hyperthermia is performed using several devices [16,17,64,65], where the choice of device used to deliver hyperthermia depends on the patient’s size, clinical opinion, and patient comfort. The BSD-2000-3D MR-compatible presents restrictive patient size guidelines [9,66], limiting the number of patients treated. Even though the imaging-based selection limits the number of patients that are amendable for reliable MR thermometry acquisition, this can be used for retrospective data selection. In addition, this study provided valuable information that gastrointestinal air motion is crucial for MR thermometry reliability. Further research on motion correction methods should also be applied retrospectively to be able to include the data corrupted by motion [67]. In recent years, new MR sequences and approaches are being studied and developed to improve MR thermometry performance and exploit the possibility to perform MR thermometry in more challenging regions [68,69,70,71,72,73,74,75]. Given the promising solutions and approaches in reach, we believe that our data will form a valuable baseline for benchmarking methods that correct internal movement [48].

### 4.3. Study Limitations

Our study had some limitations. First, we used body fat for drift correction, which was based on the fact that the proton resonance frequency of fat tissue has no frequency shift during treatment [76]. Although fat tissue contains more than 70% fat and less than 10% water, this still may result in a slight systematic underestimation of the final MR temperature. Since the temperature increase in fat tissue was small, we expect the error to be minor. Second, several techniques [67,70,71,72,77] have been developed for managing motion, such as gastrointestinal air motion, which has not been used in our study. Third, air motion was quantified by the Jaccard coefficient applied to pairs of manual segmentations. These manual segmentations were prone to intra- and inter-observer variability. Hence, we consider this a disadvantage since the air delineations were performed only by two operators, and the variability was not considered in this study. However, for hyperthermia treatment planning, an automated segmentation procedure is currently being developed at Erasmus MC; therefore, it would reduce the inter-and intra-observer variability.

## 5. Conclusions

In this study, we confirmed that air motion is a severe disturbing factor for MR thermometry and that this knowledge can be used to predict MR thermometry accuracy. We found a mean MR thermometry accuracy, precision, and bias of approximately 1 °C, 1.2 °C, and −0.3 °C, respectively, considering all locations investigated in the selected data. In addition, we showed that the overall group-mean accuracy and bias were acceptable, whereas the mean precision of the group was slightly higher than acceptable (0.2 °C). The average MR thermometry accuracy, bias, and precision were better in the selected data than the whole patient group by 50%, 80%, and 26%, respectively. Hence, our study showed that air-motion imaging-based selection before treatment predicts satisfactory MR thermometry accuracy. Therefore, this parameter has the potential to prospectively determine when MR thermometry will be valuable and might be used to replace intraluminal thermometry. In conclusion, we performed the first systematic analysis of clinical MR thermometry performance for the pelvic region. Our finding on the importance of air motion can be helpful to guide MR thermometry technology improvements. 

## Figures and Tables

**Table 1:** Characterization of the data used in this study: patient and tumor characteristics, and hyperthermia treatment sessions characteristics. For continuous data, the age, total number of sessions, and values were expressed by the mean ± standard deviation.

Characteristic	Categories	Value
Patient/Tumor Characteristics		
Total number of patients		14
Age (years)		56.5 ± 16.7
Median age (years)		60
Histology	Adenocarcinoma	3
	Squamous cell carcinoma	10
	Carcinosarcoma	1
FIGO stage	IA	1
	IB	2
	IIB	5
	IIIB	4
	IVA	2
Hyperthermia Treatment Session Characteristics		
Total number of sessions		39
Number of treatment sessions per patient		2.8 ± 1.5
Duration of each treatment session (minutes)		89.5 ± 1.6
MR thermometry scans per treatment session		8.8 ± 1.5
The time between the start of the two baseline scans (seconds)		97.0 ± 10.0
Number of MR thermometry slices with identified probes		7.3 ± 2.4
Number of probe mapping measurements during treatment time		15.2 ± 3.0
Maximum probe measurements range (cm)	Bladder	9.9 ± 2.2
	Rectum	6.9 ± 2.1
	Vagina	8.4 ± 2.5
Maximum net heating power (W)		941.1 ± 118.7

**Table 2:** Accuracy, precision, and bias parameters of MR thermometry in all treatment sessions and the selected dataset based on the Jaccard coefficient threshold equal to 0.91. All evaluation parameters are expressed by the mean (µ) ± standard deviation (σ); these mean values are also indicated in light red circles in Figure 6 and Figure 7. The number of sessions and patients remaining after exclusion are indicated. In addition, the average deviation from the acceptable threshold is given for accuracy (1 °C), precision (1 °C), and bias (±0.5 °C). The equal values or below the acceptable threshold are in boldface and underline.

	All Treatment Sessions				Selected for Air Motion (Jaccard Coefficient ≥ 0.91)			
	Bladder	Rectum	Vagina	Deviation from the Acceptable Threshold	Bladder	Rectum	Vagina	Deviation from the Acceptable Threshold
Accuracy	2.2 ± 1.6	1.9 ± 1.4	2.0 ± 1.5	+1.0 °C	1.1 ± 0.7	1.1 ± 1.1	0.9 ± 0.6	**−0.0 °C**
Precision	1.7 ± 0.9	1.6 ± 0.9	1.7 ± 0.8	+0.7 °C	1.2 ± 0.4	1.2 ± 0.6	1.3 ± 0.4	+0.2 °C
Bias	−1.5 ± 2.1	−1.2 ± 1.7	−1.2 ± 1.8	+0.8 °C	−0.4 ± 1.1	−0.4 ± 1.4	0.0 ± 1.0	**−0.3 °C**
Sessions	39 sessions (100%)				15 sessions (38%)			
Patients	14 patients (100%)				9 patients (64%)			

**Table 3:** Mean and standard deviation (µ ± σ) of temperature increase from MR thermometry measurements and intraluminal temperature measurements in delineated ROIs. The percentage of sessions and patients used in each study is compared with the total number. The mean temperature increase is reported for the dataset with low gastrointestinal air motion. MR thermometry measurements are expressed as MRT, and Intraluminal corresponds to the intraluminal measurements.

Measurements: Mean Temperature Increase (°C)						
	This Study		Other Studies			
Location	LACC		Gellermann et al. [43] Recurrent Rectal cancer		Gellermann et al. [44] Soft Tissue Sarcoma	
	MRT	Intraluminal	MRT	Intraluminal	MRT	Intraluminal
Bladder	2.4 °C ± 1.7 °C	2.5 °C ± 1.2 °C	>7 °C	No data	≤4 to 5 °C	2.6 °C ± 1.3 °C
Vagina	2.6 °C ± 1.6 °C	2.4 °C ± 1.2 °C	No data			2.2 °C ± 0.6 °C
Rectum	2.1 °C ± 1.4 °C	2.5 °C ± 1.3 °C	~3 °C			3.5 °C ± 1.0 °C
Sessions	15 sessions (38%)		15 sessions (20%)		15 sessions (50%)	
Patients	9 patients (64%)		15 patients (100%)		9 patients (100%)	

## Data Availability

The data presented in this study are available on request from the corresponding author. The data are not publicly available due to privacy.
